# There is no smoke without fire: How frequency information and the experience attribution make negative online restaurant reviews more harmful

**DOI:** 10.1371/journal.pone.0271357

**Published:** 2022-07-15

**Authors:** Wojciech Trzebiński, Beata Marciniak

**Affiliations:** Collegium of Management and Finance, SGH Warsaw School of Economics, Warsaw, Poland; Texas A&M University, UNITED STATES

## Abstract

The paper proposes and evidences that a more frequent mentioning of a service issue in an online restaurant review makes the readers blame the restaurant more for the issue. This inside attribution, in turn, may worsen the restaurant evaluation. Two experiments (Study 1 and 2) examine this mechanism using different stimuli. In both experiments, consumers exposed to high (vs. low) mentioning-frequency reviews attributed the issue more inside the restaurant and evaluated the restaurant lower. Additionally, the paper considers the role of consumer analytical processing (Study 1) and perceived review helpfulness (Study 2) in the relationships between mentioning frequency and issue attribution. The paper extends the existing literature by applying the attribution theory to the context of frequency information in online reviews. The results guide marketers dealing with negative online reviews by suggesting the way to deal with high-mentioning-frequency negative reviews.

## Introduction

Customer-generated online reviews of hospitality services, including restaurants, are omnipresent; they are provided by various opinion-sharing platforms like Tripadvisor.com, Yelp.com, and Google. Those reviews are an abundant source of information for both marketers and consumers [1–6]. The existing literature deals with the mechanisms through which online restaurant reviews influence consumer behavior, identifying various factors like temporal, sensory, and explanatory cues contained in a review, as well as its length, readability, usefulness, and enjoyability [7, 8].

Compared to expert-generated restaurant reviews, the consumer-generated ones may be perceived as more subjective [9]. This subjectivity is especially important given that restaurant service is, by definition, based on co-consumption, i.e., eating out surrounded by other visitors is a key part of the restaurant experience [10, 11]. Consequently, customers who express their opinions on restaurants in the form of online reviews may mention aspects of their experience which result not from the restaurant’s operation but rather from the activity of other visitors. For example, suppose the restaurant is visited by a group of tourists who may be too loud, make the place crowded, or leave it dirty. The negative issue (i.e., noise, crowd, or cleanliness, respectively) is an aspect of the restaurant experience that may be mentioned in the review and then attributed by the readers to those tourists or the restaurant itself. This distinction is crucial from the perspective of restaurant managers. Namely, assume consumers who read about the negative experience in an online review attribute this experience inside the restaurant (e.g., they blame it for the issue with noise, crowd, or cleanliness, respectively). In that case, their evaluation of the restaurant will be affected negatively. For this reason, investigating the determinants of the causal attribution based on online restaurant reviews may help restaurant marketers reduce the inside-the-restaurant attribution of negative experiences posted on the web, or at least reduce the possible harm resulting from this kind of attribution. The factors of causal attribution of the information presented in online reviews of hospitality services were studied, with a focus on the features of the reviewer [9, 12, 13] (Akhtar et al., 2019, Camilleri 2017, Chiou et al., 2018) or the depicted features of the service [14].

However, little is known about how this causal attribution based on an online restaurant review may be shaped by the frequency of information related to a certain aspect of a negative experience. Namely, online reviews describing the experience during a visit to a restaurant may differ in terms of the frequency of mentioning a certain issue within a restaurant experience. For example, a consumer who writes a review may share their experiences related to the noise. The review’s author may refer to that issue using only one mention (e.g., “it was definitely too loud in the restaurant”), or add more mentions (e.g., “it was very noisy around our table”, “it was much too screaming inside”, “there was a constant clattering sound in the room”). The same may be exemplified with other restaurant service issues, like crowd or cleanliness, as a review can mention each of them only once (low mentioning frequency) or multiple times (high mentioning frequency). In the latter case, the reader receives more pieces of information (arguments) indicating an issue. The lack of understanding of how that frequency information influences the way consumers attribute the issue (inside or outside the restaurant) corresponds to a vital practical problem. Namely, should managers treat high-frequency reviews differently than low-frequency ones? And specifically, how should they focus their efforts to avoid blaming the restaurant in the case of those two review types? Therefore, our study aims to bridge this gap.

**Fig 1:**
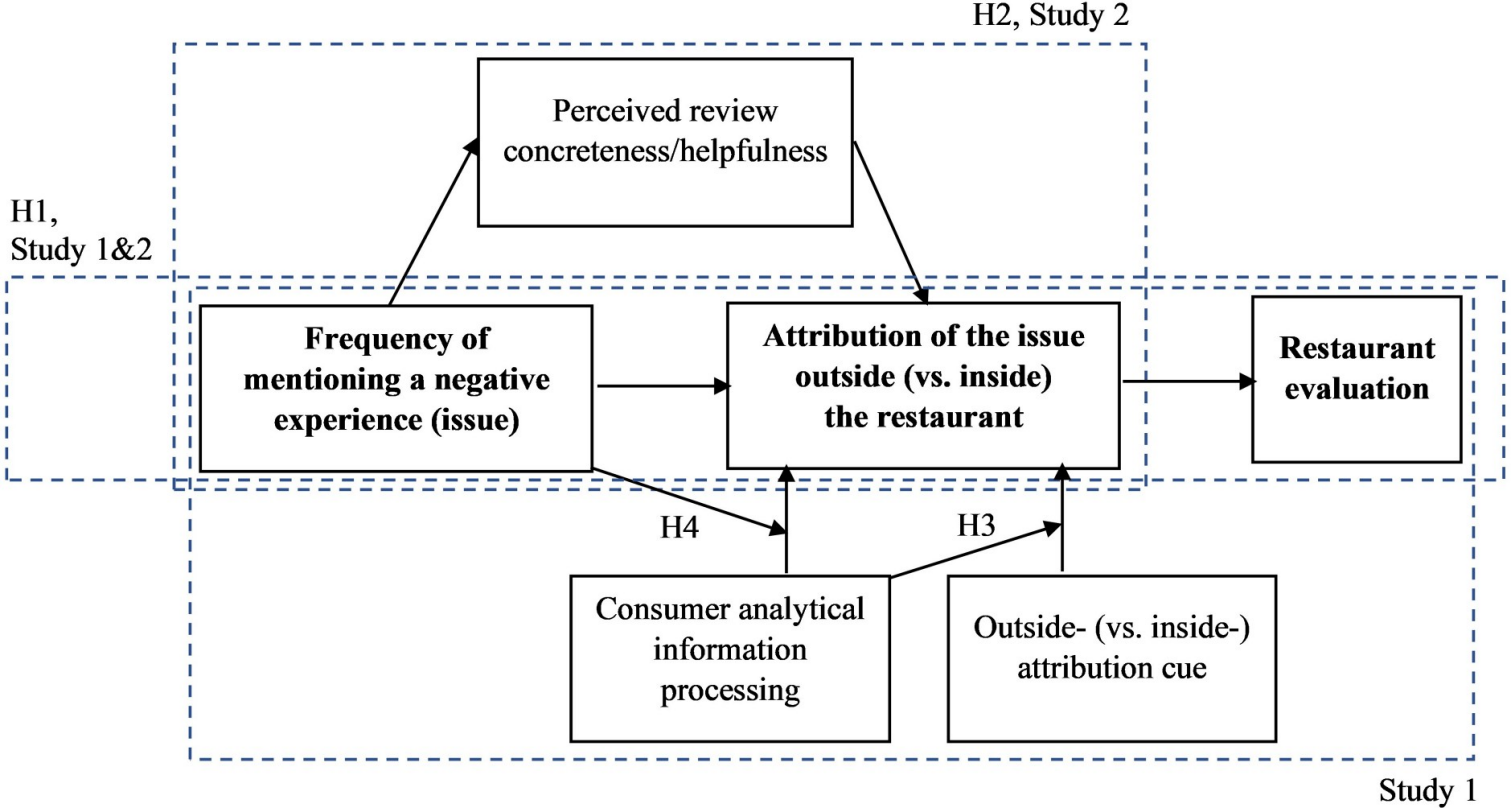
The conceptual model.

We propose that that frequency information (cf. [15]) may lead consumers who read the review to attribute the negative experience (issue) inside (vs. outside) the restaurant, even if the review contains an outside-the-restaurant explanation of the issue (outside-attribution cue). Moreover, we propose that consumers may engage their cognitive resources for analytical processing in making those attributions. We call this mechanism the frequency-information effect. Our theorization refers to the covariation-based attribution model (cf. [16, 17]), conversational norms ([18]), and message concreteness perception [19–27]. Our conceptual model, developed in the below sections, is outlined in Fig 1.

Our research adds the frequency information as a novel type of cue potentially used by consumers reacting to online restaurant reviews, enriching the existing literature on restaurant service perception (e.g., [7, 8]). We also integrate the concept of frequency heuristic ([28–30]) with causal attribution [17], conversational norms [18], and message concreteness perception (e.g., [24, 25]) in the context of online restaurant reviews.

We start with the theoretical background and the development of hypotheses, and then we examine the frequency-information effect in two experiments. By evidencing the role of frequency information in online restaurant reviews and linking it to the causal attribution of a negative restaurant experience, our paper offers a helpful reference point for service industry scholars and guides restaurant marketers in dealing with negative eWOM.

## Online restaurant reviews

The web is abundant with customer-generated reviews of hospitality services. For example, Yelp.com stored 224 million reviews as of December 2020, 18% of which were about restaurants [31]. Tripadvisor.com, which provides user-generated content related to traveling, presented more than 887 million reviews in 2021 [32]. Customer- (vs. expert-) generated restaurant reviews may be perceived by consumers as more vivid [9], as they are related to lived experiences.

In the hospitality industry, online consumer reviews are heavily researched in terms of their antecedents, like status-seeking [33], dissatisfaction [34], and consequences (e.g., [35]; see [36] for a review). Customer-generated online restaurant reviews are a source of information on the competitive advantage of restaurants [3], consumer expectations [2], and the effectiveness of marketing activities, e.g., price offers [6]. On the other hand, online restaurant reviews are researched as a factor contributing to the behavior of review readers. Kim et al. [5] demonstrated the positive relationship between the number of reviews and restaurant performance, especially when a restaurant has a certificate of excellence. In line with that, Fernandes et al. [4] demonstrated the possibility of forecasting restaurant performance using online review analytics.

The existing literature on tourism and hospitality extensively studies various forms of consumer response to online reviews, like review perceived usefulness, enjoyment, credibility, and persuasiveness [37]. Scholars investigated various factors related to the context, reader, review’s source, and the review itself. The latter group of factors includes cues related to service attributes’ ratings, a review’s length, valence, and content [37, 38]. Regarding the latter, Li et al. [7] proposed three types of cues that may influence the perceived usefulness and enjoyability of the restaurant reviews. Namely, temporal cues (i.e., a review contains time references) enhance usefulness, sensory cues (i.e., a review refers to sensory experience, e.g., taste) enhance enjoyability, and explanatory cues (i.e., a review contains explanations of reported facts) enhance both usefulness and enjoyability. Yang et al. [8] found that the review length and readability may increase the usefulness, and the presence of images may increase the enjoyability.

Of special concern to managers are the negative reviews [39], which form a substantial part of online reviews. For example, Yelp.com reports that 23% of reviews are negative (not more than two stars out of five), and the following 8% are neutral (three stars) [31]. Negative reviews are demonstrated to be more related to the behavioral outcomes as they are perceived as more diagnostic by the readers [9].

Some customer-generated online reviews focus on describing the restaurant experience. We understand the experience as various aspects of consumer emotions, thoughts, behaviors, senses [40]. Experience is what is lived by a consumer [41], and not the evaluation of a product or service itself [42]. Experiential online reviews may influence the purchase intent through the affect intensity and cognitive personalization (i.e., readers perceive the review as if it described what had happened to them) [43]. Wang et al. [44], Chen and Farn [45], and Ismagilova et al. [46] demonstrated positive relationships between certain emotions presented in online reviews and the perceived review helpfulness. In the context of restaurant reviews, Li et al. [47] found that the intensity of negative emotions expressed in the reviews was positively related to the perceived review usefulness. Based on a machine learning algorithm, Oh and Kim [48] distinguished five distinct emotions (i.e., joy, sadness, disgust, surprise, and anger) appearing in real online consumer restaurant reviews.

Experiential reviews may present information related to the restaurant visit, yet, as experience may be less objective than pure service description, the extent to which the restaurant causes the experience may be unclear to the reader. Therefore, causal attribution should be considered an important determinant of consumer response to online restaurant reviews. The existing literature investigates when certain information in reviews is attributed outside or inside the service provider. For example, Akhtar et al. [12] evidenced that the fake identity of a reviewer may increase the perception that a service failure described in the review is misrepresented. Camilleri [13] argued that a review with an outlying product rating might be discounted by readers as they make an attribution to the reviewer. Chiou et al. [9] found that consumer knowledge about a product category may increase the tendency to attribute negative reviews to the reviewer. However, in hospitality services involving a high level of co-consumption, like a restaurant or hotel, the experience is by definition shaped not only by the service provider but also by other people who co-consume the service. For example, issues with a crowd or waiting time that a reviewer reports may result from poor restaurant operation (e.g., the restaurant is providing insufficient room for the visitors or working too slowly, respectively) or from reasons outside the restaurant (e.g., other visitors arriving in a large group at once or making extensive orders that slow down the meal preparation, respectively). Therefore, consumers who read a review may attribute the experience as more or less dependent on the service provider and, consequently, perceive the provider as more or less responsible for the experience. Some studies investigate this form of attribution. For example, Browning et al. [14] demonstrated that the core aspects of hotel service (vs. staff aspects) are more likely to be attributed inside the hotel.

Despite the large volume of research on the mechanisms of consumer response to online reviews, the role of the information frequency (i.e., how many times the negative information on a certain aspect of the experience is mentioned in a review) in consumer response to the review is not fully explained. Specifically, it is not clear how the frequency of mentioning an issue in a review affects the experience attribution outside vs. inside the restaurant and what consequences it may bear on the consumer behavioral outcomes concerning the restaurant. Therefore, the present study aims to bridge this gap.

## Hypothesis development

### Frequency information and attributing the negative experience inside a restaurant

In the context of consumer online restaurant reviews, we define frequency information as the indication of how many times a certain issue (situation, event, aspect) occurred. That frequency information can be operationalized as the number of mentions of a certain aspect of a negative experience in online restaurant reviews. The consumer behavior literature widely uses the concept of frequency information or frequency heuristics, i.e., the number of mentions on a specific topic [28, 49], or the explicitly communicated number of an issue’s instances, like donations given by a company [15]. For example, product advantage may be described using only one feature or several ones. This represents the argument quantity, which is contrasted with argument quality [29, 50], also regarding online reviews [51–53]. Specifically, high-frequency information may be contrasted with high-importance information [28]. When it comes to experience descriptions, one review may mention an issue perceived as rather unimportant (e.g., noise) many times. In contrast, another review may mention some other issue only once, but that issue (e.g., waiting time) may be perceived as important in the context of the experience (e.g., the visitor was in a hurry). As consumers may attribute the experience (issue) inside the restaurant or outside of it, one may ask whether the frequency of mentioning the issue in a review may affect the experience attribution? In other words, does the number of mentions of an issue lead the consumers who read a review to consider whether the issue is a result of the restaurant’s activity or has other reasons (like the activity of other guests)?

We propose the frequency-information effect: the frequency of mentioning the negative experience (issue) leads to the attribution of the experience inside the restaurant. Firstly, the more times an attribute is mentioned in a review, the more it may be considered relevant to evaluating the restaurant itself. That “frequency heuristic” is conceptualized by Alba and Marmorstein [28] as a decision rule based on the number of positive or negative pieces of product-related information acquired by a consumer. Similarly, mechanisms of relying on the number of arguments [29] and the frequency of the event occurrence [30] were evidenced in the earlier literature. The frequency heuristic may occur when the experience is described. As this heuristic is related to making decisions regarding a product (in our case, a restaurant service), it may result in a stronger inside attribution. Namely, as more negative mentions are considered more relevant to evaluating a restaurant, the mentioned issue is perceived as more connected with the restaurant’s activity, thus leading to an inside attribution.

Secondly, in the covariation-based attribution model, the perceived covariation between a certain effect (here, e.g., an issue related to noise, crowd, or cleanliness in a restaurant) and a potential cause (here, the restaurant) is considered a key cue for attributing that effect to the cause (see review in [16, 17]). If there are more mentions of noise-related issues, these issues may be perceived by consumers as more covarying with the restaurant. Consequently, consumers may be more likely to attribute the issues inside the restaurant.

Thirdly, consumers reading an online review of a restaurant may rely on conversational norms claiming that information that is communicated is relevant [18]. Online reviews may generally be perceived by consumers as written to be a source of useful information for making decisions about products or services. Therefore, mentioning certain issues in restaurant reviews may be considered as aimed to help make decisions and, as such, firmly related to the restaurant. Hence, the more frequently a certain attribute is mentioned in a review, the more it may be interpreted as related to the restaurant, meaning that a frequently mentioned issue appears more to be a result of the restaurant’s activity.

Eventually, if an issue is mentioned more frequently in an online review, the review is likely to be more abundant in details related to the issue. Consequently, the review may be perceived as more concrete. It is heavily evidenced in the existing literature that message concreteness results in positive reactions to the message. Specifically, a more concrete message is perceived as less vague [26], more objective and truthful [23], authentic and realistic [25], vivid [20, 27], warm and competent [24], and clear [22]. Product review concreteness is related to more positive attitudes towards reviewers [19], and concrete (vs. abstract) product descriptions are more persuasive [21]. Therefore, a review that mentions a certain issue more frequently is perceived to be more concrete and, thus, may be evaluated more positively. As the general aim of product reviews is to help make decisions, this review may be assessed as more helpful. Consistently, the experience related to the frequently-mentioned issue may be perceived as more relevant to the restaurant. As such, the issue may be more attributed to the restaurant’s activity.

Causal attribution inside vs. outside a service provider may affect the attitude towards the service. When the negative experience depicted in an online restaurant review is attributed inside the restaurant, it may provide a direct negative cue for the restaurant’s evaluation, triggering negative emotions towards the restaurant as faulty [54]. On the other hand, when the attribution is outside, i.e., consumers consider the negative experience as not resulting from the activities of the restaurant, it may trigger a correction of the bias, i.e., the negative information is detached from the restaurant, and its evaluation becomes more positive [55]. Altogether, we expect the following:

**H1a.** When consumers read a restaurant review depicting a negative experience (issue), they tend more to attribute the issue inside (vs. outside) the restaurant in the case of a higher (vs. lower) frequency of mentioning the issue.

**H1b.** The attribution of the issue inside the restaurant mediates the positive relationship between the mentioning frequency and restaurant evaluation.

**H2.** The relationship between the frequency of mentioning the issue and the attribution of the issue inside (vs. outside) the restaurant (H1b) is serially mediated by perceived review concreteness and helpfulness.

### Consumer analytical processing and attributing a negative experience inside the restaurant

People may process information in two modes, coexisting on a continuum, i.e., through analytical processing, which is viewed as more effortful, controlled, logical, and rational, and automatic processing, which is depicted as less effortful, faster, intuitive, and holistic [56–58]. Consumer analytical vs. automatic information processing has been extensively studied in the context of the online environment, e.g., with regard to service seamlessness [59–61]. We propose that in the case of the inside vs. outside attribution of a negative experience depicted in an online review of a restaurant, more effortful processing may enhance an outside attribution when the related cue (e.g., “it was not clean in the restaurant after a large group of people had visited it”) is provided in the review. This is because, on the one hand, consumers may automatically consider online reviews as a relevant source of information about restaurants, implicitly assuming that experiences described in these reviews result from the activity of the restaurants (i.e., inside attribution). On the other hand, consumers may correct that automatic reasoning in the presence of an outside-attribution cue. This bias correction mechanism is demonstrated to be enhanced when people have the ability to search for potential sources of bias [55]. Thus, analytical information processing, as more effortful and controlled, may support this search while the reader is exposed to an online review. Therefore, consumer analytical processing may enhance the effectiveness of the outside-attribution cues in reducing an inside attribution of a negative experience, consequently improving the restaurant evaluation. This consideration is in line with the view by Chiou et al. [9] that more knowledgeable consumers have more cognitive resources to process review information, and therefore, they make more attributions not directly related to the product. Thus we expect the following:

**H3a.** When consumers read a restaurant review depicting a negative experience (issue), the outside-attribution cue in the description has a more positive effect on the attribution of the issue outside (vs. inside) the restaurant in the case of a more analytical (vs. automatic) consumer information processing.

**H3b.** The indirect effect of the outside-attribution cue in the description on the restaurant’s evaluation through the attribution of the issue outside (vs. inside) the restaurant is more positive in the case of a more analytical (vs. automatic) consumer information processing.

Regardless of the attribution cues, conversational norms [18] may suggest to a reader that an issue described in a negative review is relevant to evaluating a restaurant as resulting from the restaurant’s activity. Analytical information processing, related to more effortful and controlled consideration of the review’s content may make readers search for potential biases [55], including the bias related to the conventional norm of mentioning relevant information. This way, analytical information processing may increase the restaurant evaluation expressed by readers of the negative online reviews. But this mechanism may be weakened by the frequency of mentioning the issue related to the negative experience. More mentions of the issue may suggest to consumers that this experience is relevant to evaluating the restaurant itself (according to the conversational norms), as discussed above. This assessment also may consume the consumers’ cognitive resources. In line with that, previous research demonstrated that the attributional effects based on emotions were reduced when cognitive resources were limited [62]. Thus, frequency information may shift the focus of cognitive effort from bias correction to using conversational norms and consequently make the effect of analytical information processing on the outside (vs. inside) attribution less positive (or more negative). Therefore, we expect that.

**H4a.** When consumers read a restaurant review depicting a negative experience (issue), the consumer analytical information processing has a less positive (or more negative) effect on the attribution of the issue outside (vs. inside) the restaurant when the issue is mentioned more frequently.

**H4b.** The indirect effect of the consumer analytical information processing on the restaurant’s evaluation through the attribution of the issue outside (vs. inside) the restaurant is less positive (or more negative) when the issue is mentioned more frequently.

To test the above hypotheses, we ran two experiments, Study 1 and Study 2 (see Fig 1). The first experiment aimed to test H1, H3, and H4. With different stimuli, the second experiment replicates the first one and tests H2.

## Study 1

This study aimed to initially test the basic hypothesis on the frequency-information effect (H1) and test the hypotheses related to analytical information processing (H3 and H4). Study 1 drew on the aforementioned dichotomy between frequency information and importance information. Therefore, the condition of the high frequency of mentioning an issue is contrasted with the condition of the high importance of the issue. To make this contrast more realistic, we refer to two different issues related to restaurant service. The first one (i.e., cleanliness) is intended to be perceived as more complex, thus creating a better opportunity to be mentioned many times in the experience description. The second issue (i.e., waiting time) is intended to be perceived as more important. We attempted to achieve this by depicting the experience in the context of a lunch break when guests were hurrying to get back into their offices. The two above restaurant service attributes (cleanliness and waiting time) are similar as they both relate to service process of service delivery vs. the final output (i.e., a served meal) [63], and contribute more to dissatisfaction than satisfaction with the service [64]. This similarity among the attributes supports their use in the study to isolate the effect of the frequency vs. importance information provided in an online service review.

### Procedure

Two hundred thirty-two Polish young adults (aged 18–30), with at least high school education (50.4% females, M_age_ = 22.6, SD = 1.9), participated in an online experiment recruited by research assistants. Written informed consent has been collected through an online form. The design was 2 (frequency of mentioning the issue: high vs. low) × 2 (attribution cue: outside vs. inside the restaurant) × 2 (analytical information processing: high vs. low). The participants were randomly assigned to the experimental conditions. The participants were first exposed to a fictitious restaurant review in the form of a description of a visit to a restaurant. To better engage the participants, we composed the descriptions in the second person (“Imagine that you went to the restaurant…”) (see details in Appendix A). The descriptions were in the form of a story as customers write narrative reviews [65, 66] which are demonstrated to be more persuasive compared to fact-based reviews [67]. Using story-based descriptions in the stimuli allowed us to provide contextual information suggesting the high importance of waiting time in line with our setting. According to the first paragraph of the review, a group of people working together goes to a restaurant for lunch, intending to return to the office as soon as possible. This context aimed to present the waiting time in a restaurant as an important attribute of the service. The next paragraph of the review referred to the focal issue related to a negative experience at the restaurant. In the high-frequency condition, the issue was related to cleanliness, and the review provided four mentions related to the issue (i.e., “cleanliness in this restaurant left much to be desired”, “the floor was dirty”, “there were stains on tablecloths”, “chair were set up in a disorderly manner”). In the low-frequency condition, the issue was related to the waiting time mentioned in the review only once (i.e., “had to wait quite long—15 minutes”). Thus, the low-frequency condition contained importance information, which contrasted with the frequency information in the high-frequency condition. In other words, the high-frequency review provided more arguments but of low importance (“argument quantity”), while the low-frequency review provided fewer arguments but of high importance (“argument quality”). Noteworthily, in both frequency conditions, the service issue was depicted with a moderate level of extremity and intensity of the negative experience. Thus, the difference between the conditions lays in the frequency of mentions (four times in the high-frequency condition vs. one time in the low-frequency condition). Therefore, the difference focused on the amount of the issue-related detailed information provided within a review. Importantly, in both frequency conditions, the participants were exposed only to one visit description, as we aimed to examine the effect of frequency information on the response to a single review.

In the outside-attribution cue condition, the negative experience was framed as resulting from the activity of other guests at the restaurant. Namely, it was written that there was a tourist group in the restaurant, and it was suggested that those other guests were responsible for the cleanliness issue (in the high-frequency condition) or the waiting time issue (in the low-frequency condition). In the inside-attribution cue condition, it was suggested that the restaurant staff did not care about cleanliness (in the high-frequency condition) or worked slowly (in the low-frequency condition).

After depicting the negative experience, the description ended with a few pieces of information related to the waiter and the meal and suggested a mixed-valence experience (i.e., both positive and negative aspects, e.g., “the meal was tasty but not warm enough”). This part was the same for all conditions, and the aim was to make the description more comprehensive and, therefore, more realistic and engaging.

In the stimuli, the focal issue corresponded to the qualitative restaurant attributes (i.e., cleanliness and waiting time), which were generally considered to influence the restaurant evaluation more, and to increase consumer analytical processing less than quantitative attributes when consumers view a single online restaurant review [68]. This way, we aimed to make the stimuli more effective in terms of the restaurant evaluation and to maximize the effectiveness of our analytical processing manipulation. We manipulated analytical information processing after exposing the participants to the stimuli, similarly to Keller and McGill [69]. We asked the participants to provide an opinion about the restaurant. In the high analytical information processing condition, the participants were instructed to form an opinion thoroughly and to deliberate on the presented restaurant review. In the low analytical information processing condition, the participants were instructed to form an opinion quickly, based on the first thought coming to their minds.

First, the participants evaluated the restaurant. Then, we measured the analytical information processing as a manipulation check, and finally, we measured the outside vs. inside attribution. To reduce the self-generated validity issues [70], we countered our hypothesized causality [71], i.e., the outside vs. inside attribution was measured *after* measuring the restaurant evaluation. The final section of the interviews collected demographic data.

### Stimuli pretest

We conducted a pretest to check the differential perception of the two restaurant attributes corresponding to the issues included in the restaurant reviews (i.e., cleanliness and waiting time). We expected cleanliness to be perceived as more complex (i.e., consisting of more detailed aspects) than the waiting time. This would indicate that the first attribute fits well to be described with a high frequency of mentions. Secondly, we expected the waiting time to be perceived as a more important attribute of restaurant service than cleanliness. Generally, service-related restaurant attributes are perceived as more important than atmosphere-related ones [72], and this difference may be especially prominent in the context of the experience described in the stimuli. This would indicate that presenting the cleanliness with a high mentioning frequency and presenting the importance cues related to the waiting time would be realistic and compliant with the consumer perception of the restaurant service.

Two-hundred eighty-three students of marketing courses (51.6% females, M_age_ = 23.07, SD = 1.94) voluntarily participated in the pretest in exchange for course credits. Appropriate consent has been collected. The participants were randomly assigned to the experimental conditions. After reading a stimuli review, the participants were asked to rate how important the two attributes (i.e., cleanliness and waiting time) would be for them in the situation presented in the review. The importance of each attribute was rated with a single, five-point item (1 = the least important attribute, 5 = the most important attribute). The two items for the focal attributes were preceded by two filler items of waiter service and meal taste to disguise the studied relationships among the attributes and, therefore, to reduce demand bias. Following Teas [73], the participants were directed to choose “5” for the most important attribute of the four ones in the list and to choose “1” for the least important attribute. This aimed to increase participant engagement in evaluating the attribute importance. Finally, in open-ended questions, the participants were asked to list more detailed aspects of restaurant attributes (i.e., cleanliness and waiting time). The two questions regarding the focal attributes were preceded by a filler question on waiter service to disguise the studied relationships. The responses were coded by three independent raters, who were blind to the research model, and whose task was to count each distinct aspect of an attribute listed by a participant (α_cleanliness_ = .966, α_waiting time_ = .915), and scores for each attribute were pooled into a single index.

In line with our expectations, the number of the listed distinct aspects was larger for the cleanliness than for the waiting time (M_cleanliness_ = 2.006, SD = 1.264, M_waiting time_ = 1.062, SD = .857, t(282) = 13.741, *p* < .001), and this relationship occurred in both frequency conditions (high frequency: M_cleanliness_ = 2.175, SD = 1.230, M_waiting time_ = 1.126, SD = .848, t(153) = 10.294, *p* < .001; low frequency: M_cleanliness_ = 1.804, SD = 1.279, M_waiting time_ = .987, SD = .864, t(128) = 9.299, *p* < .001). Moreover, the waiting time was perceived as more important than the cleanliness (M_waiting time_ = 3.859, SD = 1.155, M_cleanliness_ = 2.922, SD = 1.461, t(282) = 7.621, *p* < .001), and this relationship occurred in both frequency conditions (high frequency: M_waiting time_ = 3.682, SD = 1.251, M_cleanliness_ = 3.065, SD = 1.440, t(153) = 3.620, *p* < .001; low frequency: M_waiting time_ = 4.070, SD = .994, M_cleanliness_ = 2.752, SD = 1.474, t(128) = 7.682, *p* < .001).

### Measurements

#### Experience (issue) attribution outside vs. inside a restaurant

Similar to Pacheco et al. [74], we used single-item measurements for causal attribution of a service failure. Specifically, the participants indicated whether they attributed the issue with the cleanliness (in the high-frequency condition) or the waiting time (in the low-frequency condition) to the restaurant. The statements were: “Considering the visit described above, the restaurant was responsible for…: …the cleanliness problems/…the problems with time of waiting for the meal.” We used a single, five-point item (1 –totally disagree, 5 –totally agree), and the scores were reversed (i.e., the higher values of the measurement indicate the issue is attributed more outside the restaurant). To disguise the studied relationships and, therefore, to reduce demand bias, we preceded this item by two similar items related to the meal and the taste, respectively.

#### Analytical information processing

We adapted the four five-point items to measure analytical information processing from Griffin et al. [75] and Smerecnik et al. [76] (e.g., “I have deliberated in detail how to respond.”), α = .840. The items were averaged into a single index. Details regarding the measurement scale are presented in Appendix B.

#### Restaurant evaluation

The participants evaluated the restaurant’s operation in terms of the issue with the cleanliness (in the high-frequency condition) or the waiting time (in the low-frequency condition). The question was: “How do you evaluate the operation of this restaurant in terms of… cleanliness/waiting time?” with a single, ten-point item (1 –“a terrible way of operating”, 10 –“an excellent way of operating”). We used a single-item measurement as we focused on a concrete aspect of the restaurant service evaluation (cf. [77]). The questionnaire pretest based on individual discussions with population study participants revealed that using ten response options (vs. five options) better enable participants to accurately reflect the level of restaurant evaluation. We used the words “operation” and “operating” to focus the measurement on the restaurant itself rather than embracing the overall experience described in the review.

### Results

#### Manipulation checks

We ran a series of ANOVAs with the experimental conditions as factors and manipulation check measurements as dependent variables. In the outside-attribution cue condition, the outside attribution of the focal issue was higher compared to the inside-attribution condition (M_outside_ = 2.340, SD = 1.144, M_inside_ = 1.650, SD = .898, F(1,224) = 29.753, *p* < .001), supporting the effectiveness of the attribution manipulation. Furthermore, in the high analytical information processing condition, the level of analytical processing was higher than for the high analytical information processing condition (M_high_ = 3.602, SD = .858, M_low_ = 3.257, SD = .904, F(1,224) = 8.696, *p* = .004), supporting the effectiveness of the analytical processing manipulation.

#### Hypothesis testing

**Fig 2:**
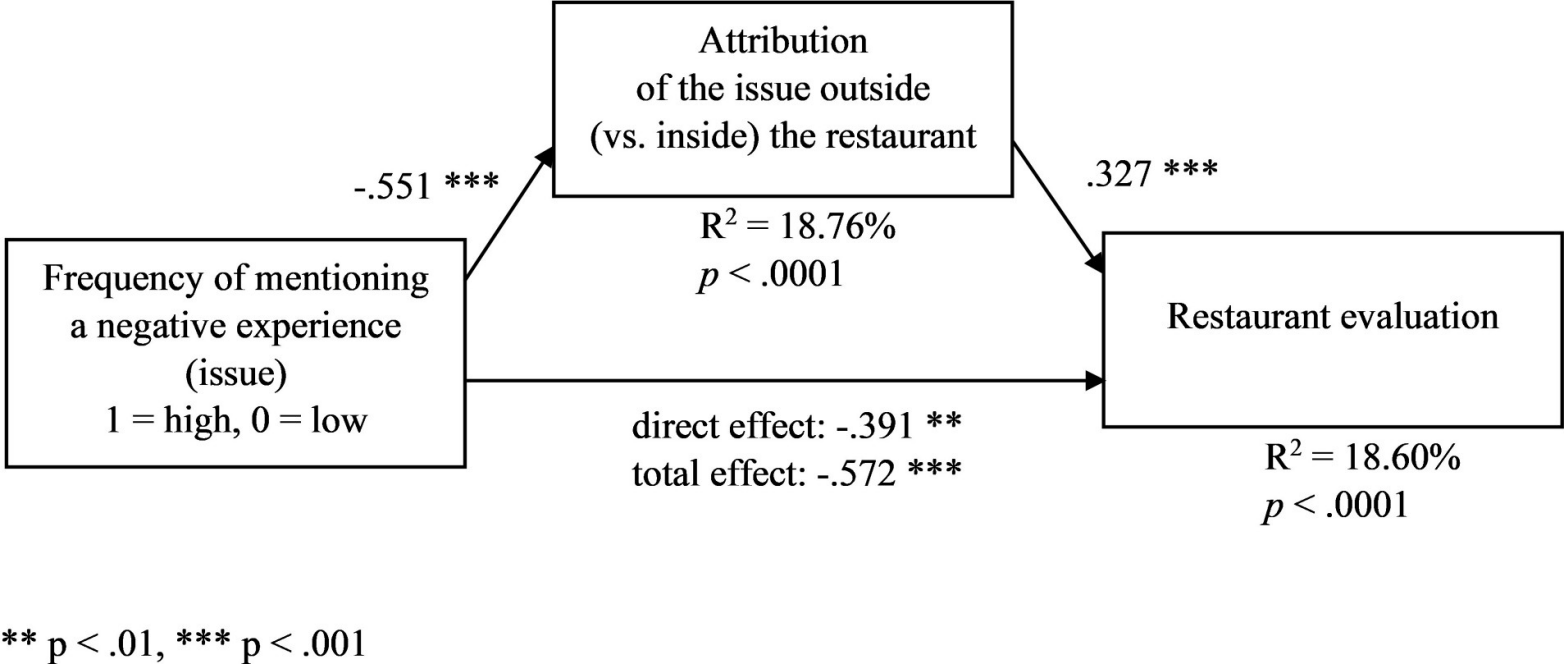
Frequency of mentioning a negative experience (issue) (manipulated), the attribution of the issue outside (vs. inside) the restaurant, and restaurant evaluation–a mediation model (Study 1). The other manipulated variables are included as covariates. ** p < .01, *** p < .001.

We ran an ANOVA with the experimental conditions as factors and outside (vs. inside) attribution as a dependent variable. In line with H1a, in the condition of high (vs. low) frequency of mentioning the issue, the outside (vs. inside) attribution of the issue was lower (M_high_ = 1.712, SD = .967, M_low_ = 2.278, SD = 1.124, F(1,224) = 20.049, *p* < .001). In other words, consumers who read the review with more mentions of the issue attributed that issue more inside the restaurant. Moreover, in line with H1b, in the mediation model with the frequency condition as an independent variable (1 = high frequency, 0 = low frequency), perceived outside attribution as a mediator, restaurant evaluation as a dependent variable, and the other manipulated variables (information processing and attribution cue) as covariates (Fig 2, PROCESS model 4, [78], VIFs < 1.3, β_total_ = -.572, *p*_total_ < .0001, β_direct_ = -.391, *p*_direct_ = .002;), the frequency had a negative effect on the outside attribution (β = -.551, *p* < .0001), while the outside attribution had a positive effect on the restaurant evaluation (β = .327, *p* < .0001), and the indirect effect of the frequency on the restaurant evaluation through the outside attribution was negative (partially standardized β = -.181, CI95%[-.291, -.094]; bootstrap sample of 5000 for all mediation analyses).

**Fig 3:**
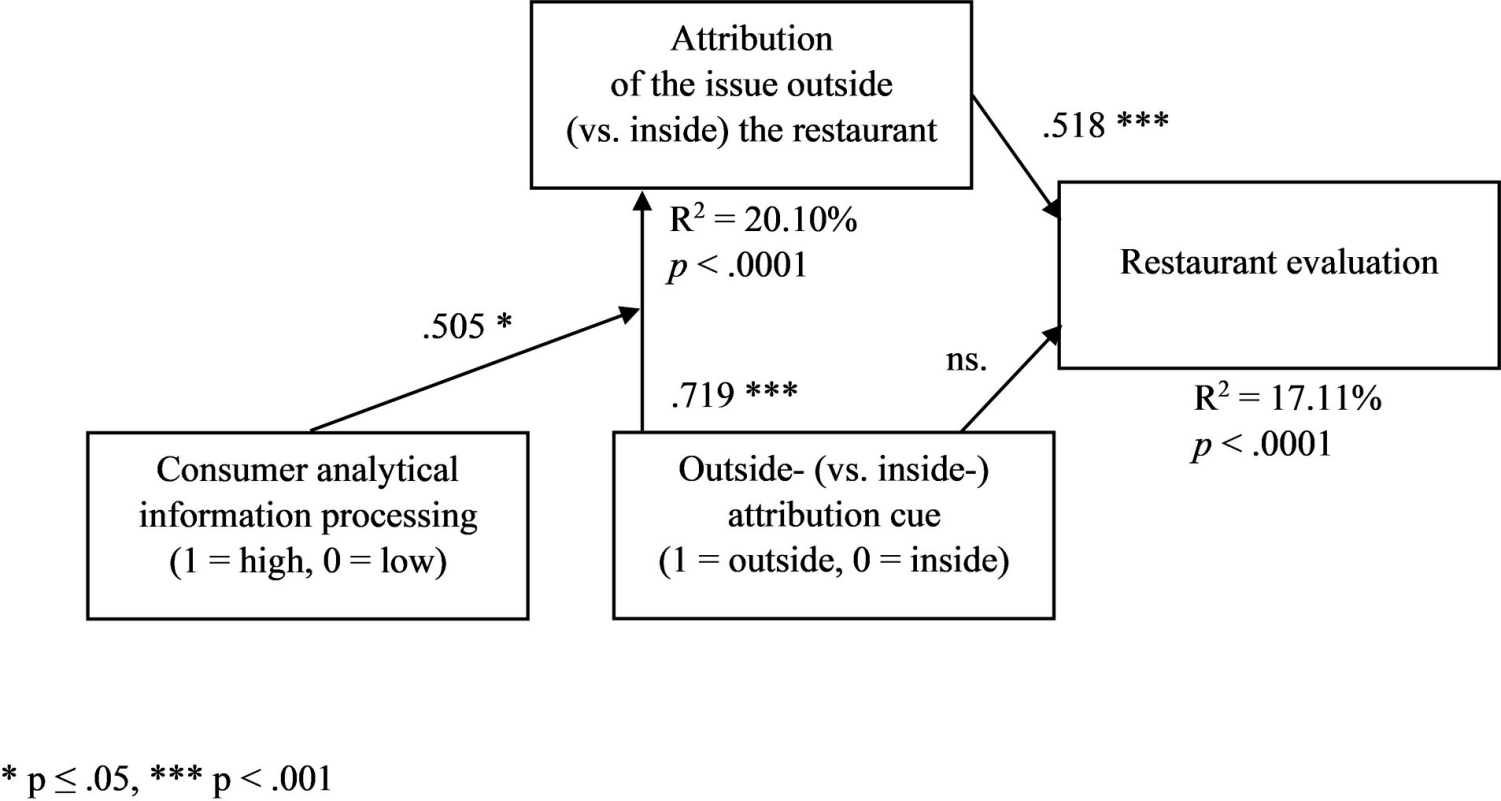
Consumer analytical information processing (manipulated) and the effect of the outside- (vs. inside) attribution cue (manipulated) on the attribution of the issue outside (vs. inside) the restaurant, and restaurant evaluation–a moderated-mediation model (Study 1). The frequency condition is included as a covariate. * p ≤ .05, *** p < .001.

In line with H3a, there occurred an interaction effect of outside (vs. inside) attribution cue and high (vs. low) consumer analytical information processing on perceived outside attribution (F(1,224) = 4.451, *p* = .036). Outside (vs. inside) attribution cue had a more positive effect on the perceived outside attribution for a higher level of consumer analytical information processing. In the high analytical-processing condition, the difference in the perceived outside attribution between outside and inside attribution cue conditions (M_outside_—M_inside_) was .957 (M_outside_ = 2.514, SD = 1.162, M_inside_ = 1.557, SD = .886). In the low analytical-processing condition, the difference (M_outside_—M_inside_) was .423 (M_outside_ = 2.166, SD = 1.099, M_inside_ = 1.743, SD = .907). Moreover, in line with H3b, the moderated-mediation analysis with the attribution cue condition (1 = outside-attribution cue condition, 0 = inside-attribution cue condition) as an independent variable, perceived outside attribution as a mediator, information processing manipulation (1 = high analytical-processing condition, 0 = low analytical-processing condition) as a first-stage moderator, restaurant evaluation as a dependent variable, and the frequency condition as a covariate (Fig 3, PROCESS model 7, [78]) revealed a significant processing-cue interaction and a moderated-mediation index (VIFs < 1.3, R^2^_change_ = .022, F_change_(1,226) = 6.147, *p*_change_ = .014; *p*_direct_ > .1, B_int_ = .505, *p*_int_ = .052; for high-analytical processing: B_indirect_ = .494, for low-analytical processing: B_indirect_ = .233; moderated-mediation index = .262, CI95%[.005, .572]).

**Fig 4:**
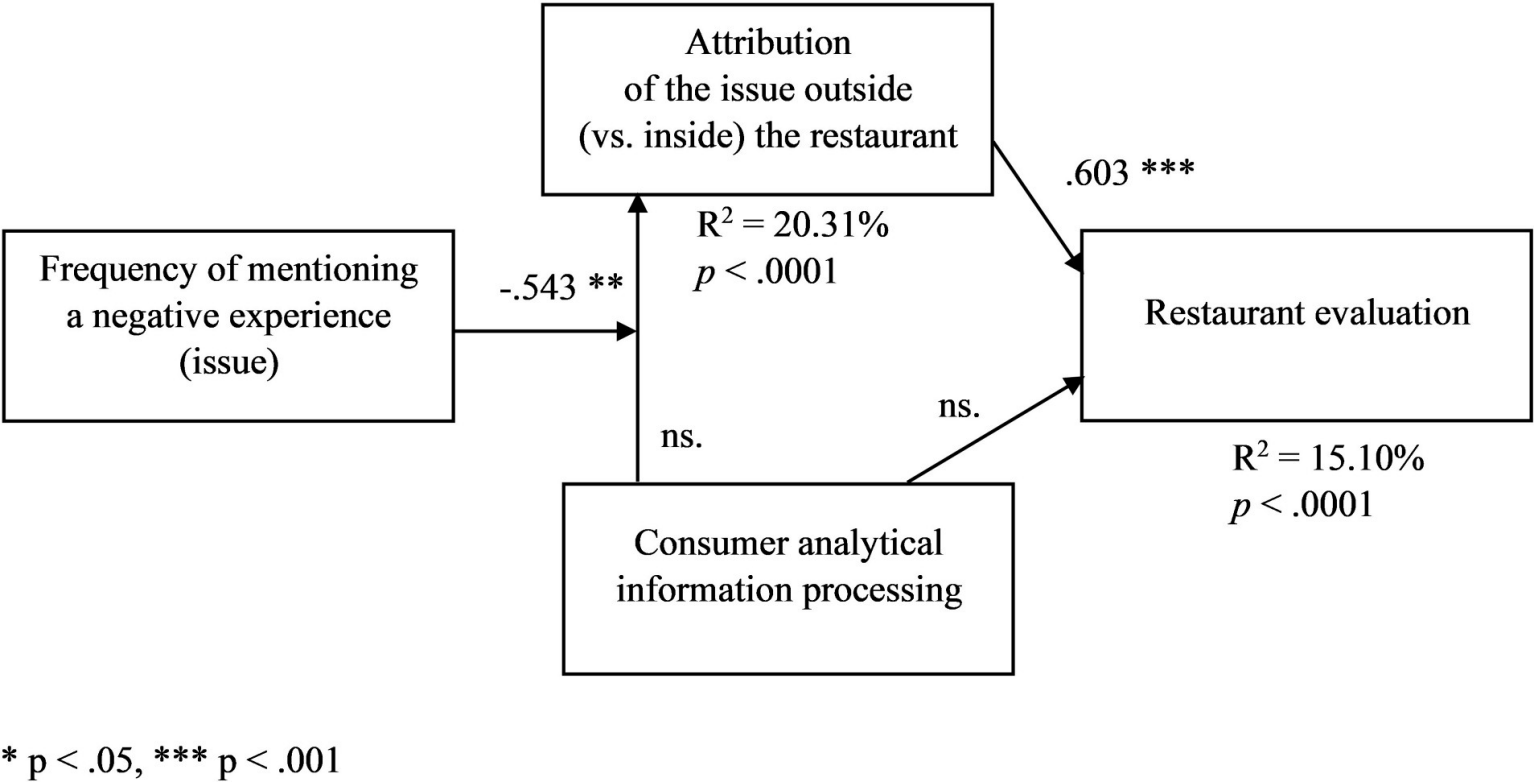
Frequency of mentioning a negative experience (issue) and the effect of consumer analytical information processing on the attribution of the issue outside (vs. inside) the restaurant, and restaurant evaluation–a moderated-mediation model (Study 1). The attribution cue condition is included as a covariate. * p < .05, *** p < .001.

Finally, in line with H4a, there occurred an interaction effect of high (vs. low) frequency of mentioning the issue and high (vs. low) consumer analytical information processing on perceived outside attribution (F(1,224) = 4.538, *p* = .034). Analytical information processing (high vs. low) had a less positive (more negative) effect on the perceived outside (vs. inside) attribution of the issue for a higher level of the frequency of mentioning the issue. In the high-frequency condition, the difference in the perceived outside attribution between the high and low analytical information processing conditions (M_high-analytical_−M_low-analytical_) was -.189 (M_high-analytical_ = 1.617, SD = .936, M_low-analytical_ = 1.806, SD = 1.001). In the low-frequency condition, the difference (M_high-analytical_−M_low-analytical_) was .350 (M_high-analytical_ = 2.453, SD = 1.172, M_low-analytical_ = 2.103, SD = 1.034). Moreover, in line with H4b, the moderated-mediation analysis with the information processing condition (1 = high analytical-processing condition, 0 = low analytical-processing condition) as an independent variable, perceived outside attribution as a mediator, the frequency condition (1 = high frequency, 0 = low frequency) as a first-stage moderator, restaurant evaluation as a dependent variable, and the attribution cue condition as a covariate (Fig 4, PROCESS model 7, [78]) revealed a significant frequency-processing interaction and moderated-mediation index (VIFs < 1.3, R^2^_change_ = .024, F_change_(1,226) = 6.859, *p*_change_ = .009; *p*_direct_ > .06, B_int_ = -.543, *p*_int_ = .037; for high-frequency: B_indirect_ = -.103, for low-frequency: B_indirect_ = .224; moderated-mediation index = -.327, CI95%[-.651, -.023]).

We also tested the effects of the two interactions (analytical information processing × attribution cue and analytical information processing × mentioning frequency) on the perceived outside (vs. inside) attribution together in one regression model. To this end, we ran a linear regression analysis with five independent variables (VIFs < 1.1): the three mean-centered experimental conditions (analytical information processing (coded as 1 for the high analytical-processing condition, 0 for the low analytical-processing condition); attribution cue (coded as 1 for the outside-attribution cue condition, 0 for the inside-attribution cue condition); mentioning frequency (coded as 1 for the high-frequency condition, 0 for the low-frequency; condition)), and the two interaction terms (analytical information processing × attribution cue and analytical information processing × mentioning frequency). The perceived outside (vs. inside) attribution served as a dependent variable (R^2^ = .217, *p* < .001). Outside (vs. inside) attribution cue had a positive effect on the perceived outside attribution (β = .324, *p* < .001), the effect of mentioning frequency was negative (β = -.271, *p* < .001; in line with H1a), the analytical information processing × attribution cue interaction effect was positive (β = .118, *p* < .046; in line with H3a), and the analytical information processing × mentioning frequency interaction effect was negative (β = -.127, *p* < .033; in line with H4a).

### Discussion of Study 1

The results of Study 1 provide initial support for the proposed effect of the frequency of mentioning an issue in a negative restaurant review on the consumers’ tendency to attribute the issue. That is, when the mentions are more frequent, the issue is attributed more inside the restaurant, and consequently, the restaurant evaluation is lower. Interestingly, in an ANOVA with the experimental conditions as factors and outside (vs. inside) attribution as a dependent variable, the effect of the mentioning frequency on the outside attribution is more negative for the outside-attribution cue condition (interaction effect: F(1,224) = 7.650, *p* = .006; for the outside-attribution cue condition: M_high frequency_ = 1.882, SD = .984 M_low frequency_ = 2.798, SD = 1.099, F(1,112) = 23.178, *p* < .001; for the inside-attribution cue condition: *p* > .1). It suggests that, even, or especially, when the context suggests an outside attribution of an issue (e.g., there was a tourist group in a restaurant and they might have caused the issue), a high frequency of the mentions (i.e., more pieces of information related to the issue) may lead consumers who read the review to blame the restaurant. Perhaps, this finding indicates a certain ceiling effect (e.g., in the presence of an inside-attribution cue, consumers tend to make inside attribution regardless of the frequency of mentioning the issue).

Additionally, Study 1 provides evidence that the positive effect of the mentioning frequency on attributing the issue inside the restaurant may lead to a worse evaluation of the restaurant evaluation. However, the mediation is only partial, which indicates that some other mechanisms triggered by the frequency may contribute to lowering the restaurant evaluation. In general, the review length influences its perceived usefulness [8]. Thus, the more arguments (i.e., mentions of an issue) are contained in the negative review, the more it is likely to be persuasive (i.e., harming the restaurant evaluation). Even in the case of outside-attribution cues, more mentions of negative experiences may induce more negative emotions, which in turn may lower the restaurant evaluation through spill-over effects [79–81]. Additionally, the meaning of the attribute in the high (vs. low) frequency condition (i.e., cleanliness vs. waiting time) might trigger different emotions (e.g., disgust vs. anxiety), and the first emotion might be more harmful for the restaurant evaluation.

Finally, the results of Study 1 provide evidence of the proposed role of consumer analytical processing in the attribution of an issue depicted in a negative restaurant review. Firstly, the results suggest that analytical processing strengthens the positive effect of the outside-attribution cue on the outside (vs. inside) attribution. In other words, consumers who have a better ability to deliberate on a review tend to use the attribution cue in the review. Secondly, the mentioning frequency seems to alter the effect of analytical processing on the attribution. Namely, analytical processing may lead more to inside attribution when the frequency is high. In other words, more mentions of an issue may lead consumers to invest their cognitive resources in deliberating on the restaurant itself., As suggested by our results, both above mechanisms influence the restaurant evaluation, as the latter is positively related to the attribution of an issue outside (vs. inside) the restaurant.

The crucial limitation of Study 1 is that it compared two kinds of service issues (i.e., cleanliness and waiting time) in high- and low-frequency conditions, respectively. To overcome this, Study 2 examines the frequency-information effect using the same service issue in all conditions.

## Study 2

The first goal of Study 2 was to replicate the frequency-information effect (H1). In Study 1, the frequency of mentioning an issue in a negative restaurant review was manipulated using different attributes of restaurant service in each condition (i.e., cleanliness served to compose the high-frequency review, while the waiting time was used in the low-low-frequency one). That setting aligned with the theoretically established distinction between argument quantity and quality. However, the two attributes’s different perceived importance and meanings might contribute to the observed relationships. That is, one may doubt if the frequency-information effect in Study 1 was actually driven by the difference in the mentioning frequency or perhaps by the difference between the two focal issues. Therefore, in Study 2, the negative issues in all stimuli reviews are related to the same focal attribute (i.e., cleanliness). Moreover, we manipulated the importance cues related to that attribute to isolate the effect of mentioning frequency from the effect of issue perceived importance. We chose cleanliness because it is perceived to be more complex (see the stimuli pretest for Study 1) and thus may provide a better opportunity to use more detailed aspects of the service experience in the high-frequency condition. Meanwhile, the perceived importance of cleanliness appears feasible to be manipulated by the context of the visit to the restaurant presented in the stimuli. Consumers may perceive restaurant cleanliness as highly important in a specific context, e.g., when they take care of another person they bring to a restaurant [82]. Similar to Study 1, we manipulated the attribution cue (outside vs. inside) to check if the frequency-information effect is visible also in when an outside-attribution cue occurs.

Secondly, in Study 2, we aimed to test H2, which predicts the serial mediation of the frequency-information effect through perceived review concreteness and helpfulness. Therefore, the scenario used in the questionnaire was directly referring to the online environment, and we measured consumer response to the review.

### Procedure

Three-hundred ten Polish young adults (aged 18–30), with at least high school education (49.7% females, M_age_ = 22.1, SD = 1.8) participated in an online experiment recruited by research assistants. Written informed consent has been collected through an online form. The design was 2 (frequency of mentioning the issue: high vs. low) × 2 (importance cue: high importance vs. low importance) × 2 (attribution cue: outside vs. inside the restaurant). The participants were randomly assigned to the experimental conditions. The participants were first exposed to a brief fictitious online review of a restaurant, which contained a description of an experience in a restaurant (Appendix A), similar to the one in Study 1. Using story-based descriptions allowed us to provide contextual information to manipulate the importance cues. In all conditions, the focal issue was related to cleanliness. In the high-frequency condition, the description provided mentions related to the issue like in Study 1 (i.e., “the cleanliness in this restaurant left much to be desired”, “the floor was dirty”, “there were stains on tablecloths”, “chairs were set up in a disorderly manner”). In the low-frequency condition, the issue was mentioned only once (“the cleanliness in this restaurant left much to be desired”). Like in Study 1, in both frequency conditions, the service issue was depicted with a moderate level of extremity and intensity of the negative experience. This way, the two frequency conditions differed only in the number of mentions related to the focal issue (i.e., cleanliness, four times in the high-frequency condition vs. one time in the low-frequency condition). Therefore, the difference focused on the amount of the issue-related detailed information provided within a review. Both frequency conditions were based on a single review, in line with our conceptual model.

In the high-importance-cue condition, the context presented at the beginning of the online review indicated that cleanliness was important to the visitors. As consumers may perceive restaurant cleanliness as highly important when they take care of another person they bring to a restaurant [82], the high-importance-cue review indicated that the visitors hosted their key client who was “pedantic” (“We knew that our client was not a consummate gourmet, but they were a pedantic person who paid a lot of attention to the cleanliness around them. Understandably, we wanted to make a good impression on them. That is why we kept our fingers crossed that everything that day in the restaurant was shining and orderly as it should be.”). In the low-importance-cue condition, the context drew attention to another restaurant attribute (“We knew that our client is not a pedantic person, but they were a consummate gourmet who paid a lot of attention to the taste of their dishes. Understandably, we wanted to make a good impression on them. That is why we kept our fingers crossed that everything that day in the restaurant was well prepared and tasted as it should.”). The context of experiencing a restaurant visit from the perspective of persons brought by the visitor is present in real online restaurant reviews; for example:

“I had a lunch in that restaurant, allegedly one of the best in that area. I had invited two guests (family) (…) It was extremely disappointing. (…) I was ashamed of having brought my guests in this restaurant.” (Tripadvisor.com)

“Had planned to take my parents for a dinner. (…) I felt shame in taking my parents to this restaurant. Wasted precious time, energy and money (…) I still can’t believe I planned to take my parents for dinner to this absolutely awful restaurant.” (Restaurant Guru)

The above examples of real restaurant reviews suggest the realism and external validity of the importance-cue manipulation based on the expectations of a person the review’s author brought to the restaurant.

We manipulated the outside vs. inside attribution the same way as in Study 1. After depicting the negative experience, the description ended with a paragraph depicting a mixed-valence experience related to a waiter and a meal like in Study 1.

After reading the review, the participants evaluated the restaurant, and we measured the outside vs. inside attribution of the issue. Then, we subsequently measured the perceived helpfulness and concreteness of the online restaurant review. This way, the sequence of our measurement countered our proposed causality to reduce the self-generated validity [71]. The interviews ended with a measurement of the perceived importance of the restaurant attributes (as a manipulation check) and a demographics section.

### Measurements

Experience (issue) attribution outside vs. inside a restaurant and the perceived importance of the restaurant service attributes were measured the same way as in Study 1, except that we used seven-point items. The larger number of response options compared to Study 1 was aimed to increase the measurement accuracy [83].

The restaurant evaluation measurement was the same as in Study 1, except that the question did not refer to any specific issue (“How do you evaluate the operation of this restaurant?”). This way, we aimed to check if the effects on restaurant evaluation demonstrated in Study 1 hold when the evaluation is not restricted to the focal issue related to the manipulation (here: cleanliness). In other words, we wanted to examine the robustness of the proposed relationships with the general restaurant evaluation as a dependent variable.

Responses to the review were measured with multi-item measurement scales. Review concreteness was measured with two seven-point items (i.e., “Assume that you really consider visiting a restaurant. If you read such restaurant review as the one presented above, you would evaluate the review as…: “…concrete”, “…detailed”; 1 –totally disagree, 7 –totally agree) similar to the existing message concreteness measurements (e.g., [84, 85], the linear correlation coefficient of the two items equaled .637.

Review helpfulness was measured with three seven-point items (., “Assume that you really consider visiting a restaurant. If you read such restaurant review as the one presented above, you would evaluate the review as…: “, helpful”, “…useful”, “…applicable”; 1 –totally disagree, 7 –totally agree), partially adapted from Huang et al. [86], α = .933.

For both these measurement scales, the scores were pooled into single indices. Details regarding the measurement scales are presented in Appendix B. To assess the discriminant validity of review helpfulness and concreteness measurements, we subjected the item scores to Exploratory Factor Analysis (EFA; Kaiser-Meyer-Olkin Measure of Sampling Adequacy (KMO) = .784, Bartlett’s *p* < .001; extraction Method: Principal Component Analysis (PCA), VARIMAX rotation). Two components had eigenvalues above 1. In support of discriminant validity, the helpfulness items loaded on the first component, and the concreteness items loaded on the second component with loadings above the .5 cut-off, and loading differences between the two components above .2 [87].

### Results

#### Manipulation checks

We ran a series of ANOVAs with the experimental conditions as factors and manipulation check measurements as dependent variables. In the high-importance cue condition, the perceived importance of cleanliness was higher than in the low-importance cue condition (M_high-importance_ = 6.162, SD = 1.421, M_low-importance_ = 5.148, SD = 1.898, F(1,302) = 28.219, *p* < .001). Next, in the outside-attribution cue condition, the outside (vs. inside) attribution of the issue was higher compared to the inside-attribution condition (M_outside_ = 3.077, SD = 1.767, M_inside_ = 1.913, SD = 1.443, F(1,302) = 39.944, *p* < .001). The above supports the effectiveness of the manipulations.

#### Hypothesis testing

**Fig 5:**
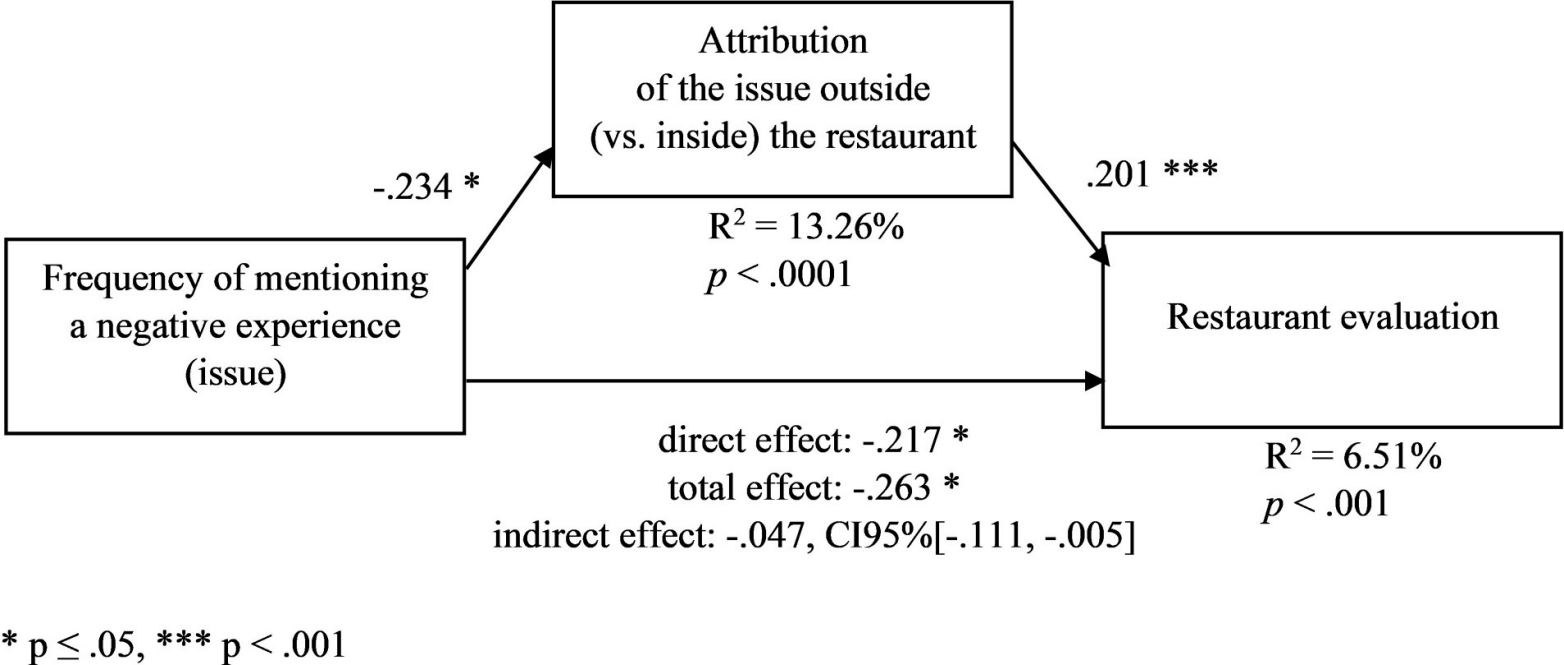
Frequency of mentioning a negative experience (issue) (manipulated), the attribution of the issue outside (vs. inside) the restaurant, and the restaurant evaluation–a mediation model (Study 2). The other manipulated variables are included as covariates. * p ≤ .05, *** p < .001.

We ran an ANOVA with the experimental conditions as factors and the perceived outside (vs. inside) attribution as a dependent variable. In line with H1a, the outside (vs. inside) attribution of the issue was lower in the condition of a high (vs. low) frequency of mentioning the issue (M_high_ = 2.298, SD = 1.620, M_low_ = 2.692, SD = 1.771, F(1,302) = 4.566, *p* = .033). Moreover, the effect of the importance cue was nonsignificant (*p* > .5). Importantly, the frequency effect was significant also in the outside-attribution-cue condition (M_high frequency_ = 2.792, SD = 1.710, M_low frequency_ = 3.363, SD = 1.781, F(1,148) = 3.893, *p* = .050). Additionally, in line with H1b, in the mediation model–with the mentioning frequency condition as an independent variable (1 = high frequency, 0 = low frequency), the perceived outside attribution as a mediator, the restaurant evaluation as a dependent variable, and the other manipulated variables (importance cue and attribution cue) as covariates (Fig 5, PROCESS model 4, [78], VIFs < 1.2, β_total_ = -.263, *p*_total_ = .020, β_direct_ = -.217, *p*_direct_ = .053)–the frequency had a negative effect on the outside attribution (β = -.234, *p* = .029), the outside attribution had a positive effect on the restaurant evaluation (β = .201, *p* < .001), and the indirect effect of the frequency on the restaurant evaluation through the outside attribution was negative (partially standardized β = -.047, CI95%[-.111, -.005]).

**Fig 6:**
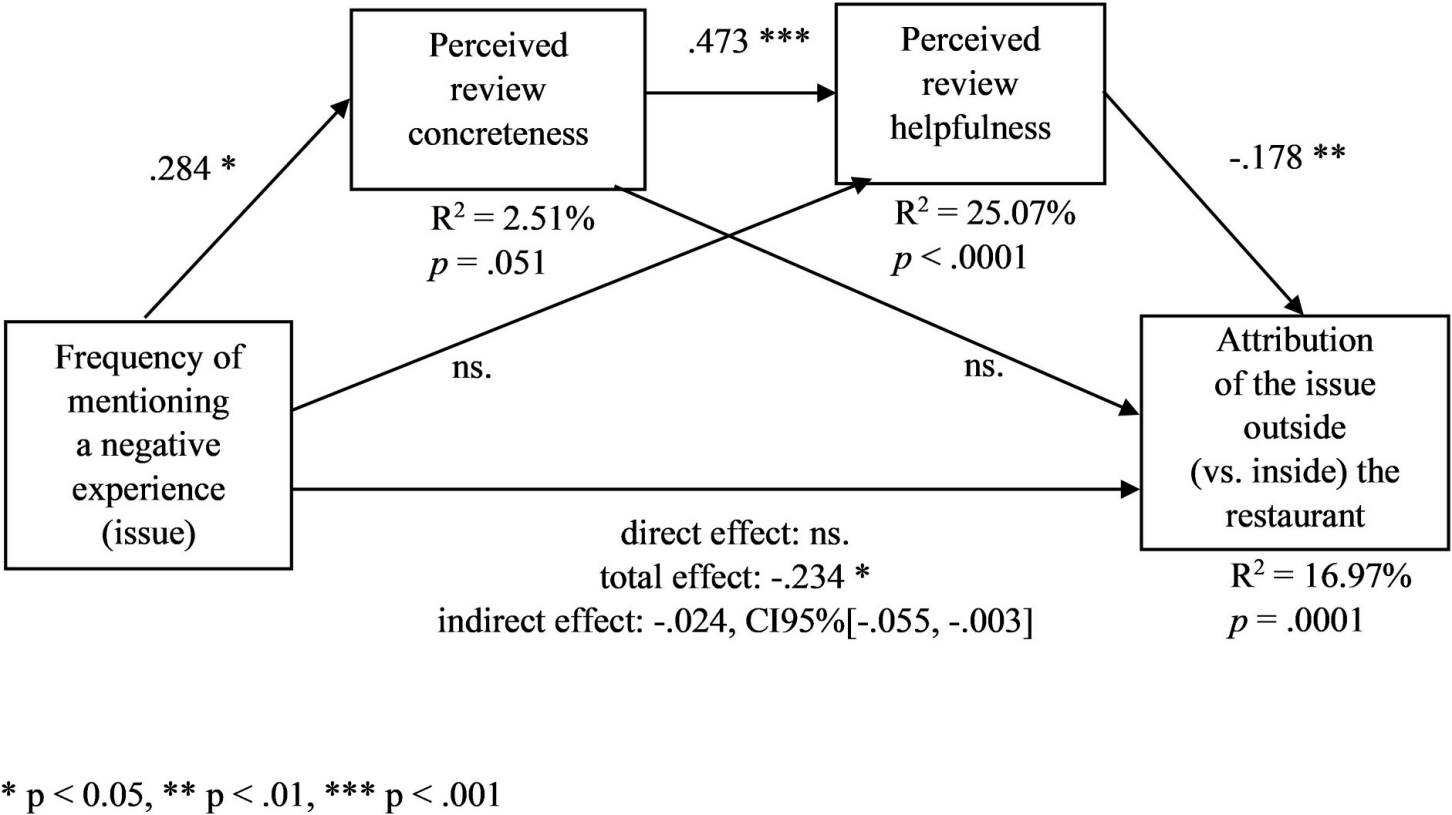
Frequency of mentioning a negative experience (issue) (manipulated), the perceived review concreteness and helpfulness, and the attribution of the issue outside (vs. inside) the restaurant–a serial mediation model (Study 2). The other manipulated variables are included as covariates. * p < 0.05, ** p < .01, *** p < .001.

Next, we ran a series of ANOVAs with the experimental conditions as factors and the perceived review concreteness and helpfulness as dependent variables. In line with H2, in the condition of a high (vs. low) frequency of mentioning the issue, the perceived review concreteness was higher (M_high_ = 5.021, SD = 1.296, M_low_ = 4.599, SD = 1.579, F(1,302) = 6.546, *p* = .011), as well as the perceived review helpfulness (M_high_ = 5.371, SD = 1.350, M_low_ = 4.959, SD = 1.526, F(1,302) = 6.250, *p* = .013). Crucially, the negative effect of the frequency on the outside (vs. inside) attribution of the issue was fully and serially mediated by the perceived review concreteness and helpfulness (Fig 6, PROCESS model 6 with the mentioning frequency condition (1 = high frequency, 0 = low frequency) as an independent variable, perceived review concreteness and helpfulness as the first and second serial mediators, respectively, the perceived outside attribution as a dependent variable, and the other manipulated variables (importance cue and attribution cue) as covariates, [78], VIFs < 1.4, β_total_ = -.234, *p*_total_ = .029, *p*_direct_ > .09). In this mediation model, the frequency of mentioning the issue had a positive effect on the perceived review concreteness (β = .284, *p* = .012), and in turn, the perceived concreteness had a positive effect on the perceived review helpfulness (β = .473, *p* < .0001), and eventually, the review helpfulness had a negative effect on the outside attribution (β = -.178, *p* = .003). The indirect effect of the frequency on the outside attribution through the perceived review concreteness and helpfulness was negative (β = -.024, CI95%[-.055, -.003]).

### Discussion of Study 2

The results of Study 2 replicate the frequency-information effect. Again, it appears that a high frequency of mentioning an issue in a negative restaurant review makes consumers who read the review attribute the issue more inside the restaurant. Importantly, in Study 2, we ruled out the possibility for this effect to be caused by the perceived importance of the issue, as the effect of importance-cue manipulation on the attribution was nonsignificant, as well as the frequency manipulation effect on the perceived importance. Moreover, as we used the same issue in both frequency conditions (i.e., cleanliness) in Study 2, we ruled out the different meanings of an issue as a possible explanation of the frequency-information effect. It even appears that isolating the frequency information from the importance information and the different meanings of an issue might enhance the effect, since the mediation of the relationship between the frequency and restaurant evaluation through experience attribution is full in Study 2 (while it is only partial in Study 1). Eventually, as the restaurant evaluation measurement did not specifically refer to the focal issue, our results provide better evidence of the frequency-information effect on the attitude towards a restaurant.

Similar to Study 1, the negative effect of the mentioning frequency on the outside (vs. inside) attribution also occurred in the outside-attribution-cue condition. In other words, even if a review directly suggested that the issue was not caused by the restaurant (but rather by a tourist group who left the place dirty), consumers reading a review with more (vs. fewer) mentions of the cleanliness issue still put the blame more on the restaurant.

Finally, Study 2 provides evidence of the frequency-information effect in detail from the online review perspective. Firstly, our findings suggest that a review with a high mentioning frequency, being perceived as more concrete, is consequently perceived as more helpful. Most importantly, though, in our mediation model, the direct effect of the frequency on the attribution is nonsignificant, which rules out the possibility that the observed relationship between the perceived helpfulness and the attribution entirely results from the influence of the attribution on the helpfulness. Namely, although the inside-the-restaurant attribution may make consumers perceive the review as more helpful, our data suggest that (at least) apart from that, the opposite relation also occurs, i.e., perceiving the review as helpful may prompt consumers to interpret the issues presented in the review as relevant to evaluating the restaurant, leading consumers to attribute these issues inside the restaurant.

## Theoretical implications

Our results advance the body of knowledge regarding the mechanism of consumers’ response to online restaurant reviews by evidencing the frequency-information effect. Namely, we demonstrate that the frequency of mentioning an issue in a negative restaurant review is positively related to perceiving the review as concrete and helpful, attributing the experience inside the restaurant, and evaluating the restaurant negatively. We show that the frequency-information effect may occur even when the review contains a direct cue of the attribution of an issue outside the restaurant. We also discern that consumers who read restaurant reviews may focus their cognitive effort on making an inside attribution based on the high frequency of mentioning an issue. This way, our research enriches the set of restaurant review perception factors investigated in the literature [7, 8] by adding the frequency information as a novel type of cue potentially used by consumers reacting to online restaurant reviews.

We also contribute to the consumer behavior theory by supporting the existing ideas linking the message type, consumer information processing, and consumer evaluation. Firstly, we provide evidence that the frequency heuristic, based on perceiving certain stimuli as occurring many times [28–30], may lead consumers who read a review to more inside-the-company attribution of the negative experience that is frequently mentioned in the review. This way, we integrate the concept of frequency heuristic with the covariance-based mechanism of causal attribution [16, 17], conversational norms [18], and message concreteness perception [19–27]. Specifically, we propose and provide empirical support for the possible mechanism in which mentioning a negative experience many times leads to perceiving a higher covariance between the restaurant and the experience, higher relevance, and helpfulness of the negative experience description, all resulting in attributing the negative experience inside the service provider and in its lower evaluation.

Secondly, we support the existing notions that the availability of cognitive resources leads consumers to correct their initial inferences [9, 55], like that the service itself causes the service failure. Namely, we apply that notion to the outside-attribution cues in online restaurant reviews and evidence that the effectiveness of these cues is higher when consumers who read the reviews process the information more analytically. Moreover, we shed new light on the above ideas showing that the effect of analytical processing on the experience attribution may depend on the frequency of mentioning a negative experience in a review. That is, when the information is more frequent, consumers use their cognitive resources to attribute the experience inside rather than outside the company.

## Practical implications

The key takeaway for restaurant managers is that negative online reviews which mention a negative experience many (vs. few) times are likely to be perceived by their readers as helpful, and the negative experience is likely to be more attributed to the restaurant’s operation. This emphasizes the potential harm to a restaurant caused by reviews of this kind. It appears that restaurants may undertake two types of activities to manage this risk. Firstly, managers may reduce the harm caused by high-frequency negative reviews. Because of the budget constraints, companies are advised to provide high-tailored responses for the most negative reviews [39]. Accordingly, restaurant managers may detect high-mentioning-frequency negative reviews using text-mining techniques (e.g., counting how many times negative experiences are mentioned in a review) and focus their efforts on responding to those reviews. Particularly, managers may attempt to discuss the real cause of a frequently-mentioned issue and, if applicable, they may point out the outside-the-restaurant causes of that issue. Potentially, managers may also attempt to reduce the frequency of mentioning negative issues. For example, considering that consumer reviews often take a narrative form [65, 66], restaurants may engage visitors in positive stories using tools like gamification. This way, the narrative which consumers have in mind after a visit may be oriented towards positive aspects, and the negative issues, if mentioned, would be depicted rather briefly.

## Limitations and further research perspectives

The effect of the frequency of mentioning an issue in a negative online review on the attribution of the issue outside (vs. inside) the restaurant should be further investigated across various types of restaurant attributes. For example, staff attributes (vs. core attributes, like cleanliness) are more likely to be attributed outside the company [14]. Therefore, it is important to check if the frequency of mentioning the staff-related issues makes consumers who read a review perceive these issues as a restaurant’s fault. Likewise, the frequency-information effect may be tested across other hospitality services (like hotels) that heavily rely on online reviews. Eventually, the results presented here should be tested among different consumer groups and cultures.

From the methodological perspective, the frequency-information effect may be tested in a field-experimental setting in online forums, e.g., based on consumer comments [9] and ratings of the review helpfulness [88]. Additionally, future studies may test the impact of high-mentioning-frequency negative reviews on restaurant performance in a similar manner as [5].

The frequency-information effect may also be studied from the perspective of its business-relevant antecedents. Specifically, future studies may investigate the antecedents of high-mentioning-frequency reviews, given that they are potentially harmful to businesses. Understanding what may lead consumers to generate reviews of that kind and what may prevent them from doing that is of high importance for restaurants and other hospitality services. For example, it should be checked whether engaging visitors in positive stories reduces the frequency of mentioning a negative experience in a consumer review.

Downstream behavioral consequences of the frequency-information effect may also be analyzed. In our paper, we focused on restaurant evaluation as a dependent variable. However, one may expect that a lowered restaurant evaluation resulting from the higher mentioning frequency of mentioning an issue may harm consumer loyalty intentions like willingness to recommend or reuse.

While our research focused on negative online reviews, a similar mechanism of the frequency-information effect may occur in the case of positive reviews. For example, a nice atmosphere in a restaurant mentioned in a review may lead its readers to attribute this positive aspect inside the restaurant even if it resulted from the activity of other visitors that were at the restaurant at the same time as the reviewer. Investigating this positive mechanism may help managers to use the opportunity provided by high-mentioning-frequency positive reviews.

## Supporting information

S1 Appendix Stimuli—Restaurant experience descriptions. (DOCX)

S2 Appendix Measurement scales used in the studies. (DOCX)
