# Supplementary material for: There is no smoke without fire: How frequency information and the experience attribution make negative online restaurant reviews more harmful
Source: PLoS One. 2022 Jul 15;17(7):e0271357. doi: 10.1371/journal.pone.0271357 (PMC9286221; doi:10.1371/journal.pone.0271357)
Supplement: S1 Appendix — (DOCX) [file pone.0271357.s001.docx]

**APPENDIX A**

**STIMULI - RESTAURANT EXPERIENCE DESCRIPTIONS**

STUDY 1

Passages depending on the experimental conditions are bolded.

frequency of mentioning the issue: high

attribution cue: outside

*Imagine that you went to the restaurant with the colleagues from your office to eat lunch. You had limited time for that, as the duties waited for you in the office. It was crucial for you to eat quickly and be able to be back in the office fast. That is why you kept fingers crossed that the staff quickly dealt with your order.*

***During your visit you noticed that the cleanliness in this restaurant left much to be desired.*** ***The floor was a bit dirty, there were stains on tablecloths in some places, some chair were set up in a disorderly manner, etc.*** ***At that moment a tourist group was dining in the restaurant. They apparently did not care about the cleanliness around them.***

*A waiter collected your order. It seemed to you that they are not so familiar with the menu. The waiter bring you your meal, asked if it was tasty, and if you want a dessert. The meal was tasty, but not warm enough. It could have been seasoned better.*

frequency of mentioning the issue: low

attribution cue: outside

*Imagine that you went to the restaurant with the colleagues from your office to eat lunch. You had limited time for that, as the duties waited for you in the office. It was crucial for you to eat quickly and be able to be back in the office fast. That is why you kept fingers crossed that the staff quickly dealt with your order.*

***After ordering your meal, you had to wait quite long - almost 15 minutes. At that moment a tourist group was dining in the restaurant.*** ***Thus, there were many orders.***

*A waiter collected your order. It seemed to you that they are not so familiar with the menu. The waiter bring you your meal, asked if it was tasty, and if you want a dessert. The meal was tasty, but not warm enough. It could have been seasoned better.*

frequency of mentioning the issue: high

attribution cue: inside

*Imagine that you went to the restaurant with the colleagues from your office to eat lunch. You had limited time for that, as the duties waited for you in the office. It was crucial for you to eat quickly and be able to be back in the office fast. That is why you kept fingers crossed that the staff quickly dealt with your order.*

***During your visit you noticed that the cleanliness in this restaurant left much to be desired.*** ***The floor was a bit dirty, there were stains on tablecloths in some places, some chair were set up in a disorderly manner, etc.*** ***Apparently, the staff did not care sufficiently about the cleanliness.***

*A waiter collected your order. It seemed to you that they are not so familiar with the menu. The waiter bring you your meal, asked if it was tasty, and if you want a dessert. The meal was tasty, but not warm enough. It could have been seasoned better.*

frequency of mentioning the issue: low

attribution cue: inside

*Imagine that you went to the restaurant with the colleagues from your office to eat lunch. You had limited time for that, as the duties waited for you in the office. It was crucial for you to eat quickly and be able to be back in the office fast. That is why you kept fingers crossed that the staff quickly dealt with your order.*

***After ordering your meal, you had to wait quite long - almost 15 minutes. You noticed that the staff worked slowly, so completing the orders took a lot of time.***

*A waiter collected your order. It seemed to you that they are not so familiar with the menu. The waiter bring you your meal, asked if it was tasty, and if you want a dessert. The meal was tasty, but not warm enough. It could have been seasoned better.*

STUDY 2

Passages depending on the experimental conditions are bolded.

frequency of mentioning the issue: high

attribution cue: outside

importance cue: high importance

*We went to the restaurant with the colleagues from your office and a key client of our company.* ***We knew that our client was not a consummate gourmet, but they were a pedantic person who paid a lot of attention to the cleanliness around them. Understandably, we wanted to make a good impression on them. That is why we kept our fingers crossed that everything that day in the restaurant was shining and orderly as it should be.***

*During our visit we noticed that the cleanliness in this restaurant left much to be desired.* ***The floor was a bit dirty, there were stains on tablecloths in some places, some chair were set up in a disorderly manner, etc. At that moment a tourist group was dining in the restaurant. They apparently did not care about the cleanliness around them.***

*A waiter collected your order. It seemed to you that they are not so familiar with the menu. The waiter bring you your meal, asked if it was tasty, and if you want a dessert. The meal was tasty, but not warm enough. It could have been seasoned better.*

frequency of mentioning the issue: high

attribution cue: outside

importance cue: low importance

*We went to the restaurant with the colleagues from your office and a key client of our company.* ***We knew that our client is not a pedantic person, but they were a consummate gourmet who paid a lot of attention to the taste of their dishes. Understandably, we wanted to make a good impression on them. That is why we kept our fingers crossed that everything that day in the restaurant was well prepared and tasted as it should.***

*During our visit we noticed that the cleanliness in this restaurant left much to be desired.* ***The floor was a bit dirty, there were stains on tablecloths in some places, some chair were set up in a disorderly manner, etc. At that moment a tourist group was dining in the restaurant. They apparently did not care about the cleanliness around them.***

*A waiter collected your order. It seemed to you that they are not so familiar with the menu. The waiter bring you your meal, asked if it was tasty, and if you want a dessert. The meal was tasty, but not warm enough. It could have been seasoned better.*

frequency of mentioning the issue: high

attribution cue: inside

importance cue: high importance

*We went to the restaurant with the colleagues from your office and a key client of our company.* ***We knew that our client was not a consummate gourmet, but they were a pedantic person who paid a lot of attention to the cleanliness around them. Understandably, we wanted to make a good impression on them. That is why we kept our fingers crossed that everything that day in the restaurant was shining and orderly as it should be.***

*During our visit we noticed that the cleanliness in this restaurant left much to be desired.* ***The floor was a bit dirty, there were stains on tablecloths in some places, some chair were set up in a disorderly manner, etc. Apparently, the staff did not care sufficiently about the cleanliness.***

*A waiter collected your order. It seemed to you that they are not so familiar with the menu. The waiter bring you your meal, asked if it was tasty, and if you want a dessert. The meal was tasty, but not warm enough. It could have been seasoned better.*

frequency of mentioning the issue: high

attribution cue: inside

importance cue: low importance

*We went to the restaurant with the colleagues from your office and a key client of our company.* ***We knew that our client is not a pedantic person, but they were a consummate gourmet who paid a lot of attention to the taste of their dishes. Understandably, we wanted to make a good impression on them. That is why we kept our fingers crossed that everything that day in the restaurant was well prepared and tasted as it should.***

***D****uring our visit we noticed that the cleanliness in this restaurant left much to be desired.* ***The floor was a bit dirty, there were stains on tablecloths in some places, some chair were set up in a disorderly manner, etc. Apparently, the staff did not care sufficiently about the cleanliness.***

*A waiter collected your order. It seemed to you that they are not so familiar with the menu. The waiter bring you your meal, asked if it was tasty, and if you want a dessert. The meal was tasty, but not warm enough. It could have been seasoned better.*

frequency of mentioning the issue: low

attribution cue: outside

importance cue: high importance

*We went to the restaurant with the colleagues from your office and a key client of our company.* ***We knew that our client was not a consummate gourmet, but they were a pedantic person who paid a lot of attention to the cleanliness around them. Understandably, we wanted to make a good impression on them. That is why we kept our fingers crossed that everything that day in the restaurant was shining and orderly as it should be.***

*During our visit we noticed that the cleanliness in this restaurant left much to be desired.* ***At that moment a tourist group was dining in the restaurant. They apparently did not care about the cleanliness around them.***

*A waiter collected your order. It seemed to you that they are not so familiar with the menu. The waiter bring you your meal, asked if it was tasty, and if you want a dessert. The meal was tasty, but not warm enough. It could have been seasoned better.*

frequency of mentioning the issue: low

attribution cue: outside

importance cue: low importance

*We went to the restaurant with the colleagues from your office and a key client of our company.* ***We knew that our client is not a pedantic person, but they were a consummate gourmet who paid a lot of attention to the taste of their dishes. Understandably, we wanted to make a good impression on them. That is why we kept our fingers crossed that everything that day in the restaurant was well prepared and tasted as it should.***

*During our visit we noticed that the cleanliness in this restaurant left much to be desired.* ***At that moment a tourist group was dining in the restaurant. They apparently did not care about the cleanliness around them.***

*A waiter collected your order. It seemed to you that they are not so familiar with the menu. The waiter bring you your meal, asked if it was tasty, and if you want a dessert. The meal was tasty, but not warm enough. It could have been seasoned better.*

frequency of mentioning the issue: low

attribution cue: inside

importance cue: high importance

*We went to the restaurant with the colleagues from your office and a key client of our company.* ***We knew that our client was not a consummate gourmet, but they were a pedantic person who paid a lot of attention to the cleanliness around them. Understandably, we wanted to make a good impression on them. That is why we kept our fingers crossed that everything that day in the restaurant was shining and orderly as it should be.***

*During our visit we noticed that the cleanliness in this restaurant left much to be desired.* ***Apparently, the staff did not care sufficiently about the cleanliness.***

*A waiter collected your order. It seemed to you that they are not so familiar with the menu. The waiter bring you your meal, asked if it was tasty, and if you want a dessert. The meal was tasty, but not warm enough. It could have been seasoned better.*

frequency of mentioning the issue: low

attribution cue: inside

importance cue: low importance

*We went to the restaurant with the colleagues from your office and a key client of our company.* ***We knew that our client is not a pedantic person, but they were a consummate gourmet who paid a lot of attention to the taste of their dishes. Understandably, we wanted to make a good impression on them. That is why we kept our fingers crossed that everything that day in the restaurant was well prepared and tasted as it should.***

***D****uring our visit we noticed that the cleanliness in this restaurant left much to be desired.* ***Apparently, the staff did not care sufficiently about the cleanliness.***

*A waiter collected your order. It seemed to you that they are not so familiar with the menu. The waiter bring you your meal, asked if it was tasty, and if you want a dessert. The meal was tasty, but not warm enough. It could have been seasoned better.*
