# Supplementary material for: There is no smoke without fire: How frequency information and the experience attribution make negative online restaurant reviews more harmful
Source: PLoS One. 2022 Jul 15;17(7):e0271357. doi: 10.1371/journal.pone.0271357 (PMC9286221; doi:10.1371/journal.pone.0271357)
Supplement: S2 Appendix — (DOCX) [file pone.0271357.s002.docx]

APPENDIX B

**MEASUREMENT SCALES USED IN THE STUDIES**

| **Construct** | **Item** | **Reliability** | **Reference** |
| --- | --- | --- | --- |
| **STUDY 1** | | | |
| **Analytical information processing** | I responded very carefully. | α = .837 | adapted from Griffin et al. (2012) and Smerecnik et al. (2012) |
|  | Before I responded, I had taken a lot of time for consideration. |  |  |
|  | I have deliberated in detail how to respond. |  |  |
|  | I paid a lot of attention to think out my responses. |  |  |
| **STUDY 2** | | | |
| **Review concreteness** | concrete | r = .637 | similar to Lee et al. (2020) and Miller et al. (2007) |
|  | detailed |  |  |
| **Review helpfulness** | helpful | α = .933 | partially adapted from Huang et al. (2020) |
|  | useful |  |  |
|  | applicable |  |  |
